# Supplementary material for: An International Non-Inferiority Study for the Benchmarking of AI for Routine Radiology Cases: Chest X-ray, Fluorography and Mammography
Source: Healthcare (Basel). 2023 Jun 8;11(12):1684. doi: 10.3390/healthcare11121684 (PMC10298418; doi:10.3390/healthcare11121684)
Supplement: Supplementary file 1 [file healthcare-11-01684-s001.zip › healthcare-2376718-supplementary.pdf]

Table S1. Details of AI models

Modality	AI model	Architectures	Dataset	Source
X-ray	Lunit INSIGHT CXR	Not provided	54 221 normal chest radiographs and 35 613 chest radiographs in patients with major thoracic diseases	[23]
	LLC "FtizisBioMed"	Not provided	Not provided	[24]
	CareMentor.AI	Inception-V3 and ResNet-50	276840 frontal X-ray images of lungs	[25]
	AI RADIOLOGY CXR	an ensemble of RetinaNet and Mask R-CNN	26,684 unique CXR	[26]
FLG	LLC "FtizisBioMed"	Not provided	Not provided	[24]
	LLC «Medical Screening Systems»	Not provided	Not provided	[27]
MMG	Lunit INSIGHT MMG	ResNet-34	31 604 cancer-positive mammograms and 19 625 benign mammograms	[28]
	LLC «Medical Screening Systems»	an ensemble of deep ultra-precise neural networks combined with an aggregation module.	Not provided	[29]

Table S2. Diagnostic performance metrics per AI model

Modality	AI model	AUROC (CI 95%)	Sensitivity* (CI 95%)	Specificity* (CI 95%)
X-ray	Lunit INSIGHT CXR	0.94 (0.87-1.0)	0.90 (0.79-1.0)	0.89 (0.79-0.98)
	LLC "FtizisBioMed"	0.77 (0.66-0.88)	0.83 (0.69-0.96)	0.71 (0.58-0.85)
	CareMentor.AI	0.88 (0.81-0.96)	0.76 (0.60-0.91)	0.91 (0.82-0.99)
	AI RADIOLOGY CXR	0.87 (0.80-0.95)	0.83 (0.71-0.94)	0.89 (0.80-0.98)
FLG	LLC "FtizisBioMed"	0.89 (0.83-0,96)	0.79 (0.68-0.91)	0.92 (0.84-0.99)
	LLC «Medical Screening Systems»	0.82 (0.73-0,91)	0.71 (0.58-0.84)	0.90 (0.81-0.98)
MMG	Lunit INSIGHT MMG	0.96 (0.92-0,99)	0.83 (0.73-0.94)	0.96 (0.91-1.0)
	LLC «Medical Screening Systems»	0.81 (0.72-0.89)	0.62 (0.48-0.76)	0.92 (0.84-0.99)

* at the operating point of maximum Youden index
